# Biomechanical characteristics of different methods of neo‐chordal fixation to the papillary muscles

**DOI:** 10.1111/jocs.17027

**Published:** 2022-10-13

**Authors:** Luis Fernández, Amparo Martínez Monzonís, Mohammad M. El‐Diasty, Carmen Álvarez‐Lorenzo, Ángel Concheiro, Ángel L. Fernández

**Affiliations:** ^1^ Department of Applied Physics, School of Physics University of Santiago de Compostela Santiago Spain; ^2^ Division of Cardiology University Hospital Santiago de Compostela Spain; ^3^ Division of Cardiac Surgery Kingston General Hospital Kingston Canada; ^4^ Department of Pharmacology, Pharmacy, and Pharmaceutical Technology University of Santiago de Compostela Santiago Spain; ^5^ Divison of Cardiac Surgery, Department of Surgery, University Hospital University of Santiago de Compostela Santiago Spain

**Keywords:** chordal replacement, mitral valve repair, polytetrafluoroethylene

## Abstract

**Background and Aim of the Study:**

Several techniques have been described for neo‐chordal fixation to the papillary muscles without any reported clinical differences. The objective of this study is to compare in vitro the biomechanical properties of four of these common techniques.

**Methods:**

We studied the biomechanical properties of expanded polytetrafluoroethylene neo‐chordal fixation using four techniques: nonknotted simple stitch, nonknotted figure‐of‐eight stitch, knotted pledgeted mattress stitch, and knotted pledgeted stitch using commercially available prefabricated loops. Neo‐chordae were submitted to a total of 20 traction‐relaxation cycles with incremental loads of 1, 2, and 4 N. We calculated the elongation, the force‐strain curve, elasticity, and the maximum tolerated load before neo‐chordal failure.

**Results:**

The elongation of the neo‐chordae was lowest in the simple stitch followed by the figure‐of‐eight, the pledgeted mattress, and he commercially prefabricated loops (*p* < .001). Conversely, the elastic modulus was highest in the simple stitch followed by the figure‐of‐eight, the pledgeted mattress, and the prefabricated loops (*p* < .001). The maximum tolerated load was similar with the simple stitch (28.87 N) and with the figure‐of‐eight stitch (31.39 N) but was significantly lower with the pledgeted mattress stitch (20.51 N) and with the prefabricated loops (7.78 N).

**Conclusion:**

In vitro, neo‐chordal fixation by nonknotted simple or nonknotted figure‐of‐eight stitches resulted in less compliance as opposed to the use of knotted pledgeted stitches. Fixation technique seemed to influence neo‐chordal biomechanical properties, however, it did not seem to affect the strength of the suture when subjected to loads within physiological ranges.

**Abbreviations**
ePTFE: expanded polytetrafluoroethylene
J: Joule
N: Newton

## 1 INTRODUCTION

Mitral valve repair by using artificial expanded polytetrafluoroethylene (ePTFE) neo‐chordae has been proven to be a safe and durable technique.^1^, ^2^, ^3^ The use of ePTFE neo‐chordae either with free‐hand technique or with premeasured neo‐chordae (loop technique) has contributed to the increasing rates of mitral valve repair in patients with degenerative mitral regurgitation.^1^, ^2^, ^3^

The use of ePTFE material offers some advantages over other types of nonabsorbable suture materials. Its softness, minimal memory, and greater compliance result in minimal tissue trauma when the knots rub against the mitral leaflets surface. Also, it has a high resistance to mechanical stress and a good performance under repeated traction.^4^, ^5^

The ePTFE has microfine porosity with 50% air volume and, therefore, the diameter of the suture is reduced when subjected to traction leading to increased stress.^4^, ^6^ On the other hand, this porous microstructure facilitates tissue integration, which can contribute to reducing mechanical stress and increasing its durability.^7^

It is generally recommended to attach the artificial neo‐chordae to the fibrous portion of the head of the papillary muscle to minimize injury to the muscle tissue. There are multiple stitching techniques that have been described to anchor the ePTFE neo‐chordae to the papillary muscles^8^ such using a simple nonknotted stitch,^9^, ^10^, ^11^ a figure‐of‐eight stitch,^12^, ^13^, ^14^ a mattress stitch reinforced with a single unknotted pledget^15^, ^16^ and a mattress stitch reinforced with two pledgets knotted over the papillary muscle.^17^, ^18^, ^19^, ^20^

In vitro studies have shown that both the knotted mattress stitch with two pledgets and the figure‐of‐eight stitch provided adequate tensile strength within the limits of normal physiological range.^21^, ^22^ However, it is possible that the technique of fixation may also determine other biomechanical properties of the artificial neo‐chordae such as compliance and rigidity.

The purpose of this work is to study in an in vitro model the effect of the different fixation techniques on the biomechanical properties and the performance of ePTFE neo‐chordae.

## 2 MATERIALS AND METHODS

Four neo‐chordal fixation techniques were studied; the nonknotted simple stitch, the nonknotted figure‐of‐eight stitch, the knotted mattress stitch with two pledgets, and a pledgeted stitch using commercially available prefabricated ePTFE loops.

For the simple and the figure‐of‐eight stitches, a CV5 ePTFE suture (W.L. Gore & Associates Inc.) was used. Neo‐chordae of 4.4 cm length were constructed by knotting a CV‐5 ePTFE suture on a 2.8 cm diameter polypropylene mandrel as previously described.^23^

For the pledgeted mattress stitch, a CV5 ePTFE suture and two 6 × 5 × 1.5 mm PTFE pledgets were used. A mattress stitch was performed by knotting two ePTFE pledgets over a steel hook of 3 mm diameter. The hook was then removed, and a 4.4 cm long neo‐chorda was created following similar steps as described above.

For the premeasured loops, commercially available loops of 24 mm length knotted on the upper surface of a PTFE pledget were used (Implant Chordae Loop®, Santec GmbH).

**Figure 1:**
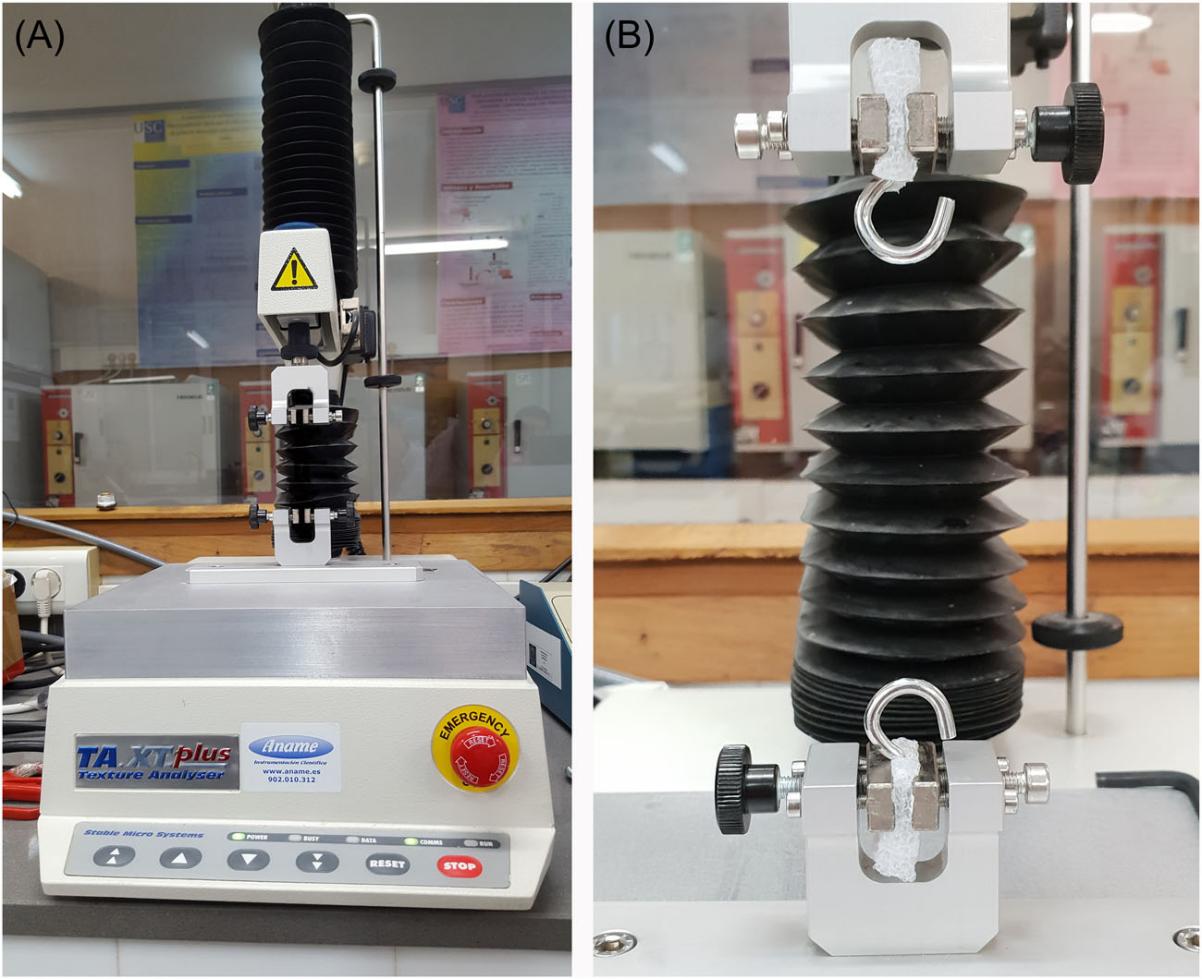
(A) Computer‐controlled texture analyzer. (B) Hooks fixed to the upper and lower clamps of the texture analyzer

A computer‐controlled texture analyzer (TA.TX Plus; Stable Micro Systems Ltd.) was used to study the mechanical properties of the different neo‐chordal fixation configurations. Steel hooks were fixed to the upper and lower clamps of the texture analyzer (Figure 1).

**Figure 2:**
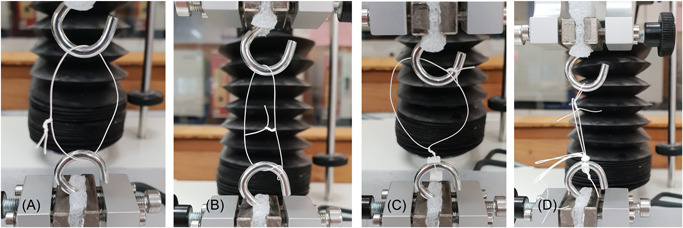
Different modes of fixation. (A) Nonknotted simple stitch. (B) Nonknotted figure‐of‐eight stitch. (C) Knotted mattress stitch with two pledgets. (D) Commercially prefabricated loop

All sutures were soaked in 0.9% saline for 10 min before dynamic evaluation which was performed at room temperature. Neo‐chordae were hooked around the two steel hooks as demonstrated in Figure 2. The prefabricated Implant Chordae Loop® was secured with a mattress stitch on one of the hooks using a second pledget to support the suture. The distal end of one of the loops was fixed to the other hook by means of a simple stitch of CV5 ePTFE (Figure 2).

A total of 10 neo‐chordae fixed as a simple stitch, 10 neo‐chordae fixed as a figure‐of‐eight stitch, 10 neo‐chordae anchored with a pledgeted knotted mattress stitch and 10 prefabricated loops (Implant Chordae Loop®) were studied.

After mounting the neo‐chordae onto the steel hooks, traction‐relaxation cycles were performed by applying rhythmical widening and narrowing of the gap between these hooks The experiments were carried out under cycle until count mode, which consisted in recording the force‐strain curves of the suture when subjected to uniaxial tension at a rate of 0.5 mm/s until a maximal predetermined force of 1 N for 20 cycles of traction‐relaxation (video S1). The same suture was further subjected to 20 more cycles of traction‐relaxation applying a maximal force of 2 N. The same process was finally repeated using a force of 4 N.

For the purposes of calculating the strain produced at each level of load, we excluded the readings from the first two traction‐relaxation cycles. This is to account for the preconditioning of the neo‐chordae which is defined as the irreversible lengthening of the suture that occurs when the knots are tightened due to traction.^24^

Since each neo‐chordae consists of a loop with identical arms, it can be assumed that the traction force generated by the texture analyzer is equally distributed over the two arms. Therefore, the magnitudes of forces applied to each arm were in fact 0.5, 1, and 2 N, respectively. Of note, these values lie within the physiological range of loads to which native chords in the adult human being are exposed.^25^, ^26^, ^27^

The average maximum elongation was calculated over 18 cycles of traction‐relaxation for each force magnitude and fixation technique. The force data were converted to stress (N/mm^2^) and the mean elastic modulus for each force and fixation method was calculated as the slope of the stress‐strain curve.

Finally, neo‐chordae were subjected to increasing tensions at a rate of 0.5 mm/s until failure defined as suture rupture or knot unraveling. The tensile force and the elongation at failure were recorded as well as the energy accumulated during traction defined as the area under the force/elongation curve. Maximum resistance was also studied for each of the four fixation techniques.

### 2.1 Statistical analysis

Continuous variables are expressed as mean value ± standard deviation. Statistical analysis was performed by the R‐Studio software (RStudio Team [2016]. RStudio: Integrated Development for R. RStudio, Inc.). The normal distribution of data was checked by the Kolmogorov–Smirnov test. One way analysis of variance following by Scheffé test were used to determine whether differences existed in the mean values. A probability value of less than .05 was considered statistically significant.

## 3 RESULTS

During each of the traction‐relaxation cycles, neo‐chordal elongation followed by shortening was observed in all the fixation models.

Elongation during traction was proportional to the applied force, however, significant differences were observed between the four neo‐chordal fixation techniques with each of the three loads.

Elongation was less observed with the simple stitch configuration and progressively increased with the figure‐of‐eight stitch, pledgeted mattress stitch and reached its maximum level with the prefabricated loop.

Table 1 shows the mean values of maximum elongation depending on the fixation mode of the neo‐chordae and the load applied during traction.

**Table 1:** Value of maximal elongation during stretching according to the neo‐chordal fixation technique and load force of traction

Maximal elongation (%)					
Force (N)	Simple stitch	Figure‐of‐eight	Pledgeted mattress	Prefabricated loop	*p* value
1 N	2.58 ± 0.27	4.4 ± 0.78	6.46 ± 0.88	9.78 ± 2.68	<0.001^a^
2 N	3.22 ± 0.28	5.83 ± 1.12	8.66 ± 1.03	12.54 ± 2.7	<0.001^a^
4 N	4.09 ± 0.35	7.24 ± 1.13	11.37 ± 0.49	17.23 ± 3.20	<0.001^a^

*Note*: Elongation (%). N = maximal load force in Newton.

^a^ Statistically significant between all fixation techniques.

**Figure 3:**
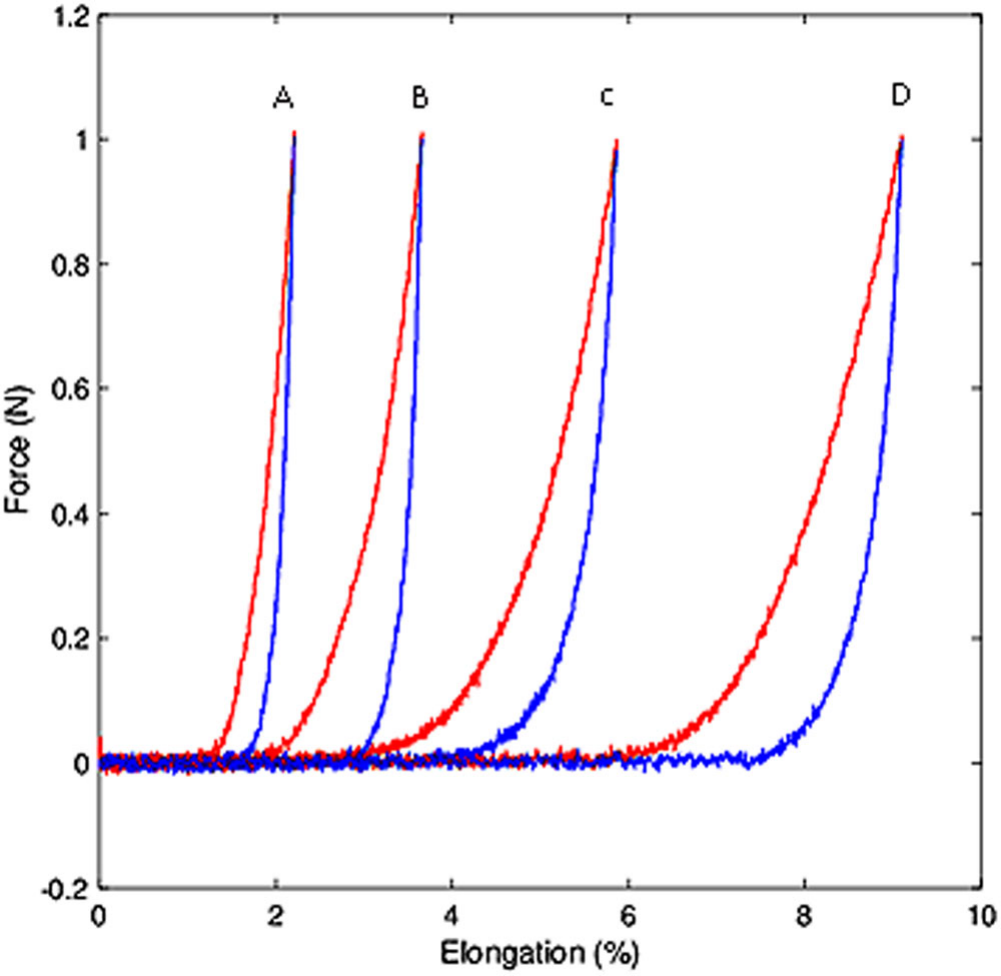
Average force‐elongation curve for 18 cycles of uniaxial loading (red)/unloading (blue) for a maximal load force of 1 N in the four models of neo‐chordal fixation. A: Simple stitch. B: Figure‐of‐eight stitch. C: Pledgeted mattress stitch. D: Prefabricated loop

The morphology of the force‐strain curve was similar for the three tensile loads. A more linear response was observed during traction (stretching) of the neo‐chordae than during recovery (shortening) upon cessation of traction. This difference between force/strain during loading versus unloading represents the mechanical hysteresis. It was also observed that the separation of the stretch curve from the relaxation curve increased progressively from the simple stitch to the figure‐of‐eight stitch to the pledgeted mattress stitch and reached a maximum with the prefabricated loop. On the other hand, the stretch‐relaxation curve showed a tendency to flatten from the simple stitch to the figure‐of‐eight and the pledgeted stitches. This shows a progressive reduction in stiffness, with the prefabricated loop being the least rigid and the one with the most hysteresis. Figure 3 shows the force‐strain curve for an applied load of 1 N in the four neo‐chordal fixation models.

It is observed that for all applied tensile forces, the elastic modulus is greater in the simple stitch and progressively decreases with the figure‐of‐eight stitch, the pledgeted mattress stitch and the prefabricated loop, with the differences between the four configurations being statistically significant. Table 2 shows the values of the elastic modulus for the different methods of neo‐chordal fixation. These findings indicate that the stress sustained by the suture is maximum with the simple stitch due to its greater stiffness and minimum with the prefabricated loop due to its greater compliance.

**Table 2:** Value of mean elastic modulus according to the neo‐chordal fixation technique and load force of traction

Elastic modulus (N/mm^2^)					
Force (N)	Simple stitch	Figure‐of‐eight	Pledgeted mattress	Prefabricated loop	*p* value
1 N	2867 ± 314	1614 ± 195	974 ± 125	999 ± 77	<0.001^a^,^b^
2 N	5148 ± 577	3064 ± 388	1809 ± 175	1713 ± 129	<0.001^a^,^b^
4 N	8484 ± 842	5683 ± 677	3276 ± 295	2467 ± 148	<0.001^a^,^b^

*Note*: N = maximal load force in Newton.

^a^ Statistically significant when comparing simple stitch vs. figure‐of‐eight stitch.

^b^ Statistically significant when comparing nonpledgeted vs. pledgeted stitches.

Regarding the maximum tolerated load before neo‐chordal failure, it was observed that all the models could safely resist forces at least ten times higher than the physiological range.

In the simple and figure‐of‐eight models, the mode of neo‐chordal failure was suture rupture at the level of the knot without any observed knot unraveling. In the pledgeted mattress stitch, suture rupture took place at the level of the knot located at the opposite end to the pledgets. Interestingly, with the commercially prefabricated loop, suture rupture did not occur, and failure was caused by sliding of the knotted loops on the PTFE pledget (video S2).

It was found that the simple and the figure‐of‐eight stitches tolerated maximum loads higher than the pledgeted mattress stitch. Regarding the maximum elongation values at the time of suture failure, the pledgeted mattress stitch presented greater elongation than the simple and the figure‐of‐eight stitches. In the analysis of maximum load, elongation, and absorbed energy, the prefabricated loop was not included because its mode of failure was different. Table 3 shows the tensile load values at the time of suture failure, suture elongation, and energy accumulated in the suture for each of the fixation techniques.

**Table 3:** Value of load force, elongation, and energy at failure for each neo‐chordal fixation technique

Force (N)	Simple stitch	Figure‐of‐eight	Pledgeted mattress	Prefabricated loop	*p* value
Load (N)	28.87 ± 2.33	31.39 ± 2.48	20.05 ± 1.79	7.78 ± 0.24^a^	<0.001^b^
Elongation (mm)	5.14 ± 0.79	6.53 ± 0.84	10.31 ± 0.5	NA	<0.001^b^
Energy (J)	0.062 ± 0.016	0.069 ± 0.014	0.049 ± 0.005	NA	ns

*Note*: N = load at failure in Newton. Elongation = maximal elongation in mm. J = energy in Joule.

^a^ Load to sliding of the knotted loops on the prefabricated Implant Chordae Loop®

^b^ Statistically significant when comparing nonpledgeted vs. pledgeted stitches.

Regarding the energy accumulated in the suture up to the moment of rupture, no differences were observed between the fixation methods.

**Figure 4:**
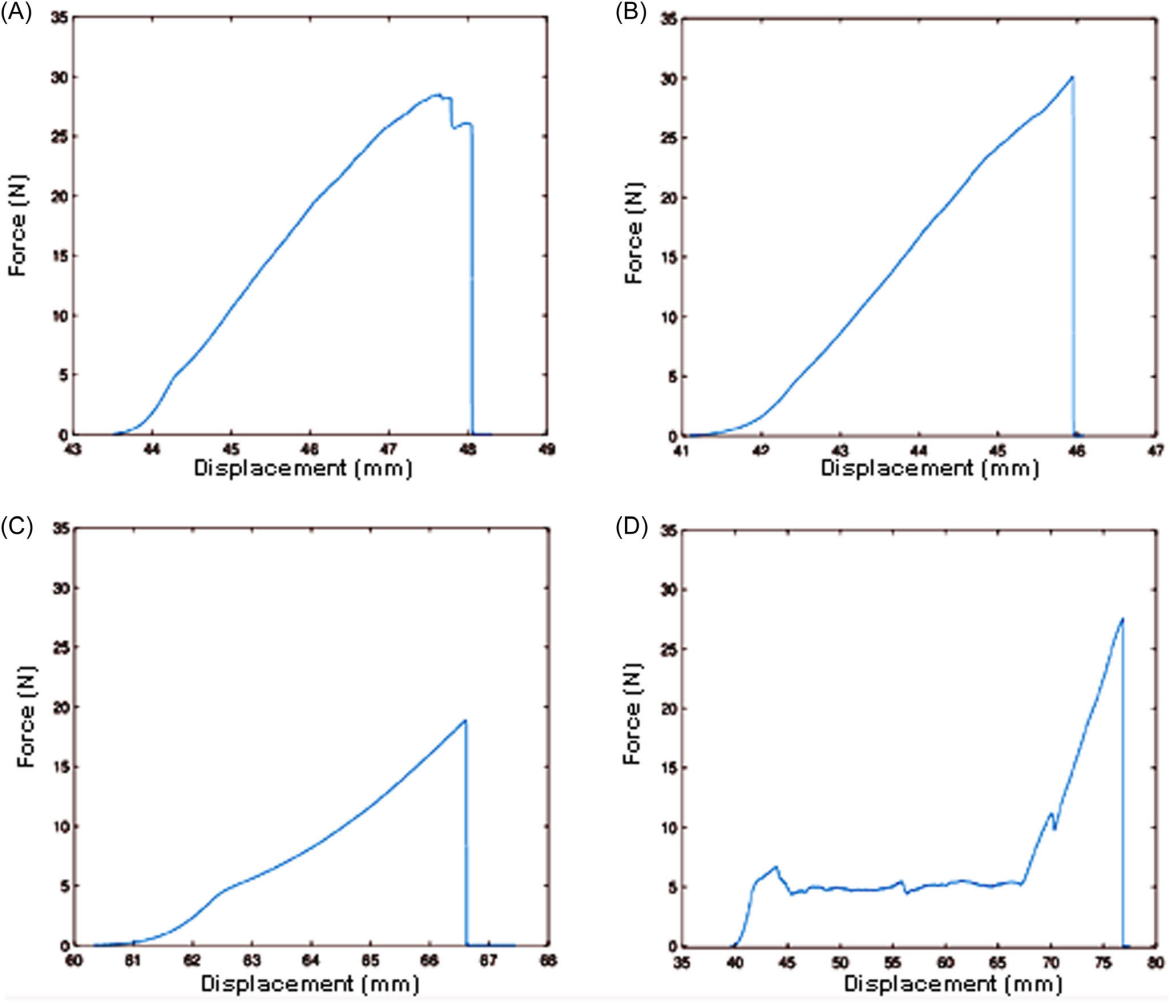
Average force‐displacement curve to suture failure. The area under curve represents accumulated energy. (A) Simple stitch. (B) Figure‐of‐eight stitch. (C) Pledgeted mattress stitch. (D) Prefabricated loop

The force‐elongation curve until suture failure of the prefabricated loop has a special morphology. It was observed that starting at 7 N of traction force, the knotted loops slipped over the PTFE pledget. Once the four loops have all slipped, if the traction continues, the suture ruptures at loads of 25–30 N at the level of the knot of the CV5 ePTFE suture used to fix the loop to the hook (video S2). Figure 4 represents the force‐elongation relationship until rupture for each of the different fixation methods.

## 4 DISCUSSION

Determining the appropriate height is probably the most critical step of neo‐chordal implantation techniques and it has been the focus of many published studies.^8^, ^28^ Variable techniques have been described to attach the neo‐chordae to the papillary muscles, however, the differences between these techniques have not been thoroughly investigated.^28^

Some authors suggest that using a simple nonpledgeted nonknotted stitch allows the two arms of the suture to automatically adjust to equal length^10^ while others suggest that a pledgeted nonknotted U‐stitch may allow for some adjustment in length by the sliding the of suture over the pledget.^16^ These two techniques may offer an advantage by equally distributing the tension on both arms of the neo‐chordae.

Some in vitro studies reported similar resistance to rupture between neo‐chordae fixed by means of a nonknotted figure‐of‐eight stitch and the prefabricated loops knotted on the surface of a PTFE pledget and anchored via a mattress stitch reinforced by a second pledget.^21^ The advantage of the figure‐of‐eight stitch could be the less burden of foreign material and the potential lower risk of infective bacterial endocarditis.^21^

However, to date no clinical or experimental studies have been published on the effect of different fixation techniques on the biomechanical properties of ePTFE neo‐chordae. For the first time, we studied in vitro the effect of four different fixation techniques on some biomechanical properties such as elongation, stress, elasticity, and resistance to rupture.

We were able to demonstrate that the simple and the figure‐of‐eight stitches have greater rigidity, lower hysteresis, and less elongation despite that in theory they should allow for some degree of sliding of the sutures on the papillary muscle and consequently a more uniform distribution of tension between the two arms of the loop.^10^, ^16^

On the contrary, the more complex fixation techniques such as the pledgeted mattress stitch and the prefabricated loop were found to be more distensible and showed a biomechanical behavior similar to that of the native chordae. It has been suggested that this characteristics may contribute to a higher resistance to fatigue and rupture.^5^, ^24^

Several factors can explain why the mode of fixation may explain the differences in the biomechanical behavior of the neo‐chordae.

First, in the mattress stitch and the prefabricated loop, traction is applied on a greater number of knots that are also supported on porous pledgets. This can result in the increase in the internal visco‐elastic damping during each cycle as well as an increase in hysteresis, favoring greater compliance and less rigidity.

Second, it has been observed that the longer an ePTFE suture is subjected to uniaxial traction the more rigid it becomes. This increase in stiffness when uniaxial traction is applied could be due to a greater internal friction force^5^ as well as a possible whipping phenomenon.^29^ The simple stich reproduces with great accuracy the uniaxial traction model. On the contrary, in the figure‐of‐eight stitch and the pledgeted stiches the transmission axis of the applied force is modified by the different throws of the suture. This change in the traction axis can potentially modify the conformation of the chemical bridges of the ePTFE, making it more compliant.

In relation to the resistance to rupture test, all the neo‐chordal fixation techniques were stable even when subjected to loads significantly higher than the physiological range, therefore they seemed to be equally reliable to use.

While in the simple stitch and the figure‐of‐eight stitch neo‐chordal rupture occurred at the knot level, it was observed that in pledgeted mattress stitch rupture occurred at the level of knots located at the opposite end to the pledgets. This is in line with previous findings that the decrease in the diameter of the suture at the level of the knots as well as the possible change in the conformation of the chemical bridges of the ePTFE can cause an increase in stress on the knots, resulting in rupture when traction is applied.^6^, ^30^

It should be noted that the simple stitch and the figure‐of‐eight stitch, despite being less compliant, support greater maximum load until rupture than the pledgeted stitches. This behavior is apparently contradictory since, in general, the higher the stiffness, the greater the possibilities of fatigue and rupture of the material.^24^ To explain this contradictory behavior, it is important to differentiate between two types of the ePTFE suture rupture; the acute rupture due to sudden exposure to supra‐physiological loads and the delayed rupture due to chronic suture fatigue resulting from the exposure to repeated physiological loads. On one hand, acute rupture occurs in absence of chronic fatigue of the suture material and the breaking strength depends, not only on the material stiffness, but also on the symmetrical distribution of the load and the reduction in the diameter of the suture at the level of the knots. On the other hand, delayed suture rupture occurs due to chronic stress from repeated traction at physiological loads. In this case, chronic suture fatigue may result in vulnerable areas of material microfractures and calcification where rupture may occur. This may explain why, in the long‐term, a higher rate of rupture occurs in the sutures whose configuration entails increased stress.

In this study, sutures were exposed to acute supraphysiological load without time to develop chronic fatigue: The simple and the figure‐of‐eight sutures resisted a greater maximum load despite being more rigid, probably because of their configuration that facilitates uniaxial traction with a more uniform load distribution, thus preserving the structure of the ePTFE and providing it with greater maximum resistance.

Regarding the premeasured loop, it was observed that starting at a tensile force 10 times higher than the physiological range, the sliding of the knotted loops on the PTFE pledget occurred. It was also observed that the suture gradually elongated as the loops were sliding. In the force‐displacement curve, this behavior is represented as a flat line that remains constant until the fourth and last loop is untied. If the traction force continued to increase, rupture occurred at the level of the CV5 ePTFE suture knot at the opposite end of the loop.

In relation to the mode of neo‐chordal fixation in the free edge of the leaflet, numerous techniques have also been described without any proven superiority of any of these techniques over the others. Some authors suggest implanting the neochords at the free edge of the prolapsed leaflet at the site of maximal prolapse while others prefer the thickened point of the leaflet where the original native chord was attached.^8^, ^28^ Regarding the knotting technique for neo‐chordal fixation in the free edge of the leaflet, the use of pericardial or ePTFE pledgets has been described if the leaflet tissue seems fragile, as well as the use of clips and polypropylene stiches to prevent sliding of the ePTFE knots.^8^, ^28^ Most authors use the figure‐of‐eight or the surgeon's knot to prevent the sliding of the knots when tying the ePTFE suture in the free‐hand technique.^8^, ^28^ The simple stitch may be, however, more appropriate to anchor the premeasured loop to the free edge of the leaflet. The objective of our study was to assess the biomechanical behavior of different neo‐chordal fixation techniques to the papillary muscle, therefore we elected to apply the same fixation technique to the leaflet edge (simple stitch) to avoid adding more confounding variables to the study.

## 5 LIMITATIONS

This study has several limitations.

First, the fixation of the sutures to the texture analyzer was performed on rigid components that are different in terms of biomechanical properties from papillary muscles and valve leaflets.

Second, only 20 traction‐relaxation cycles were performed, which does not allow to study the long‐term behavior of the different models. Previous experimental models demonstrated that by increasing the number of traction‐relaxation cycles, the ePTFE tends to become more rigid.^24^ Also, the speed of the traction‐relaxation cycles was significantly lower than that occurring during the human cardiac cycle. This difference can potentially result in changes in biomechanical behavior as it was suggested that increasing the traction‐relation speed may lead to microfractures of the ePTFE which can affect its resistance. Similarly, the frequency of the traction‐relaxation cycles can affect the behavior and viscoelastic properties of ePTFE.^29^

Finally, for the calculation of the elasticity modulus, the load force data were converted to stress by dividing the applied load force by the initial cross‐sectional area of the suture, assuming that there were no changes in the cross‐sectional area when force was applied. This assumption was made since all sutures are made of the same material and, therefore, the change in cross‐sectional area with traction should be consistent among the different fixation models.

## 6 CONCLUSIONS

In vitro, neo‐chordal fixation to papillary muscles by means of nonknotted simple stitch or nonknotted figure‐of‐eight stitch resulted in greater rigidity and less compliance as opposed to the use of knotted pledgeted stitches.

At an experimental level, the technique of fixation was shown to influence neo‐chordal biomechanical properties, however, it did not seem to significantly affect the strength of the suture when subjected to loads within physiological ranges.

More clinical and experimental studies are required to further identify the most adequate neo‐chordal fixation technique for mitral valve prolapse repair.

## AUTHOR CONTRIBUTIONS

*Concept and design*: Angel L. Fernández, Carmen Alvarez‐Lorenzo, Amparo Martínez. *Data analysis and interpretation*: Luis Fernandez, Carmen Alvarez‐Lorenzo. *Drafting article*: Carmen Alvarez‐Lorenzo, Angel L. Fernández, Angel Concheiro. *Critical revision of article*: Amparo Martínez, Mohammad El‐Diasty, Angel L. Fernández. *Approval of article*: Luis Fernández, Amparo Martínez, Mohammad El‐Diasty, Carmen Alvarez‐Lorenzo, Angel Concheiro, Angel L. Fernández. *Statistics*: Luis Fernandez, Mohammad El‐Diasty.

## CONFLICTS OF INTEREST

The authors declare no conflicts of interest.

## Supporting information

Video 1. Stress–strain cycle in simple stitch, figure‐of‐eight stitch, pledgeted mattress stitch and prefabricated loop.

Video 2. Failure test in simple stitch, figure‐of‐eight stitch, pedgeted mattress stitch and prefabricated loop.
